# Multi-objective optimization model for dynamic airspace allocation of urban drones in medical emergency delivery

**DOI:** 10.3389/fpubh.2025.1727647

**Published:** 2026-02-12

**Authors:** Yao Zhu, Xin Sun, Tongdi Hou

**Affiliations:** Business School, Yancheng Polytechnic College, Yancheng, China

**Keywords:** dynamic airspace allocation, emergency medical services, multi-objective optimization, UAV emergency delivery, urban air mobility

## Abstract

**Introduction:**

Urban emergency delivery systems face serious challenges in dealing with traffic congestion, structural limitations, and accessibility challenges that confront traditional ground-based emergency medical services. This study proposes a novel multi-objective optimization approach to the dynamic airspace allocation for unmanned aerial vehicles (UAVs) in urban emergency environments.

**Methods:**

The approach simultaneously optimizes four competing objectives including the decrease in delivery time, the costs, the increase in airspace usage efficiency, and the reduction in risks incurred by hazards to safety. An Improved NSGA-III (Non-dominated Sorting Genetic Algorithm III) algorithm with real-time adaptability characteristics and conflict resolution algorithms for emergency conditions was developed. The effectiveness was evaluated through in-depth Monte Carlo simulations that applied real urban emergency scenarios from the metropolitan area of Hangzhou, taking measurements from a metropolitan area of 150 km^2^ with 35 tertiary hospitals, among different UAV fleet configurations.

**Results:**

Simulation results suggest considerable performance improvements, with a decrease in response time by 61% (from 18.5 to 7.2 min), as well as a decrease in delivery costs by 85% (from $280 to $42.3) when compared to calibrated ground-based emergency services baseline. The dynamic reallocation mechanism provided 85 to 96% reference performance in adverse conditions such as mass incidents, adverse weather, and equipment failures, with adaptation cycles achieved in average time of 1.8 ± 0.3 s. Sensitivity analyses revealed that the size of the UAV fleet emerged as the primary performance-influencing factor.

**Discussion:**

This simulation-based framework provides a foundation for future emergency response system improvements, with pilot trials and hardware-in-the-loop testing representing essential next steps for practical deployment. The findings have considerable policy implications for urban air mobility planning and resource allocation.

## 1 Introduction

The increasing urbanization and growing complexity of metropolitan areas have posed significant challenges to traditional emergency medical service (EMS) delivery systems, particularly in time-critical scenarios where ground-based transportation faces substantial limitations due to traffic congestion, road infrastructure constraints, and accessibility barriers. Unmanned aerial vehicles (UAVs) have emerged as a transformative technology in emergency healthcare delivery, offering unprecedented capabilities to overcome these geographical and logistical obstacles while providing rapid response times essential for life-saving interventions (1, 2). The integration of UAV technology into medical emergency systems has demonstrated substantial potential for revolutionizing healthcare logistics, enabling the delivery of automated external defibrillators, blood products, medications, and other critical medical supplies to remote or inaccessible locations with significantly reduced response times compared to conventional ground vehicles.

However, the widespread deployment of UAVs in urban environments for medical emergency delivery requires sophisticated airspace management systems capable of handling multiple competing objectives while ensuring safety, efficiency, and regulatory compliance. Current urban airspace design concepts primarily focus on maximizing safety and capacity with limited consideration for the dynamic nature of emergency scenarios and the complex trade-offs between multiple operational objectives (3, 4). The challenge becomes particularly acute when considering the heterogeneous nature of UAV fleets, varying payload requirements, battery constraints, and the need for real-time adaptation to changing emergency demands and environmental conditions (5). Furthermore, the multi-objective nature of urban UAV operations necessitates balancing economic optimization with environmental and social considerations, as recent research has highlighted the importance of comprehensive cost frameworks that extend beyond traditional economic metrics (6).

This research addresses the critical gap in dynamic airspace allocation for urban medical emergency delivery by developing a novel multi-objective optimization model that simultaneously considers delivery time minimization, operational cost reduction, airspace utilization efficiency, and safety risk management. The proposed framework incorporates real-time environmental monitoring, adaptive allocation mechanisms, and conflict resolution strategies specifically designed for emergency scenarios where traditional static allocation approaches prove inadequate (7). The primary contributions include the development of a comprehensive mathematical model for dynamic airspace allocation, the design of an enhanced multi-objective optimization algorithm with real-time adaptation capabilities, and the provision of extensive experimental validation through realistic urban emergency scenarios.

## 2 Literature review

### 2.1 UAV applications in medical emergency services

The past half-decade has seen a remarkable upswing in the use of drones for emergency medical transport, a trend spurred by trial after trial that repeatedly demonstrate unmanned craft can outrun traditional supply convoys in rugged terrain. In Ghana, Damoah et al. (8) documented one such experiment in which AI-guided quadcopters carried blood products and pharmaceuticals between clinics. That endeavor burned almost no fossil fuel and still allowed local entrepreneurs to pocket a modest fee for the service. According to the pilots’ own statistics, the airborne lockers cut patient waiting times from weeks on a dirt road to under 60 min. Yet a systematic review conducted by De Silvestri et al. (9) surfaced a confused agenda of roadblocks that continue to stall broad national deployment. Regulators are demanding formal environmental impact statements, while insurers insist that cargo boxes be literally crash-proof before they will underwrite commercial flights. In the interim, most of the high-profile successes remain eye-catching press releases rather than routine hospital procedure.

Recent studies have zeroed in on dispatch support platforms that sit above bespoke optimization engines designed for the breakneck tempo of medical drone operations. Eichleay et al. (10) built a toolkit that allows logistics planners to calculate the precise payload capacity of a single UAV, information that helps public health managers sidestep emerging transport chokepoints. In parallel, Maciass et al. (11) examined the economic angle and assembled a tactical guide for slotting drones into the supply loops of inner-city hospitals; they concluded that up-front expenditure drops dramatically compared with standard truck deliveries. A distinct line of inquiry by Otero Arenzana et al. (12) focused on the hospitals’ own concourses, where refined packing heuristics demonstrated, with on-site data, that UAVs can trim expenses while still meeting care benchmarks. Robakowska and her co-authors then chronicled the patchy journeys—mishaps, victories, and everything in between—that finally bridged the gap between mathematical modelling and street-level reality. The application in practice is further detailed by Robakowska et al. (13) who evaluated the feasibility in the application of UAVs for pre-hospital interventions for both medical emergencies, with the realization that careful planning in respect to the coordination and risk evaluation protocol is essential.

The recent technological progress has seen the evolution towards ever-more advanced applications for drones in the medical sector. Therefore, researchers have explored the application of artificial intelligence-based methodology in conjunction with blockchain security solutions. Sarvesh et al. (14) proposed a framework for a network for emergency medical material, focusing on a decentralized autonomous organization paradigm, with the objective of limiting the vulnerabilities in security and risks for intercepting data that characterize traditional drone systems. Gowsalya and Mayuri (15) outlined the revolutionary aspects of drone delivery systems powered by artificial intelligence in green supply chain healthcare, using mathematically formulated equations with a focus on optimizing costs, efficiency in time, emissions, and energy consumption. These proposals are supported by a study done by Sanz-Martos et al. (16), which conducted a systematic evaluation confirming that unmanned aerial vehicles increase victim location tasks dramatically, aid in early triage processes, and ultimately promote the quality in patient care compared to traditional procedural processes.

### 2.2 Urban airspace management for UAVs

Managing low-altitude city skies for drones is far from routine; the mix of buildings, no-fly zones, and on-the-go demand makes every minute a fresh maths problem. Early momentum came from Kopardekar et al. (17), who sketched an Unmanned Aircraft System traffic-management playbook built around a simple-if-hard risk yardstick. Their motto—flexibility where possible, structure where essential—now pins itself to nearly every PowerPoint slide on urban air lanes. A more recent leap landed in ElSayed and Mohamed (18), who married real-time 3-D city modelling with a dynamic four-dimensional grid and wound up squeezing another 10 per cent of flying room out of a block of sky while also cutting trajectory energy use by half.

Designing three-dimensional corridors in city skies is no longer just about squeezing more traffic into a defined tube; it now demands a city-wide view that includes gridlock management and systemic resilience. Stuive and Gzara (19) met that bigger picture head-on by building an airspace-network model that picks the leanest slices of a given transport grid for vertical flight paths while running a constrained-optimum assignment to gauge how smoothly those lanes will flow under battery and congestion caps. Their work, and the real-world runs that followed it, underscored how closely money limits must hug shifting traffic peaks and the hard ceiling on route detours if unmanned networks are to function as anything more than a prototype. Cramming extra vertical layers on top of one another also means weather bursts, noise rules, and fire-code spreads have to get baked into the maths upfront, lest safety margins evaporate the moment a half-dozen mission types cross the same block of sky in half an hour.

### 2.3 Multi-objective optimization in UAV operations

Researchers drawn to the unruly complexity of UAV flights have, almost by instinct, begun tinkering with multi-objective optimization methods that try to keep dozens of conflicting targets in the air at once. Abdel-Basset et al. (20) roll out a fresh batch of trajectory-tuning routines aimed squarely at fleets propping up mobile edge-computing nodes. Their trick the team calls a cyclic-crossover-and-adaptive-population mash-up outperformed older, one-goal routines whenever the simulation logs were checked. The paper goes one step further by insisting that any serious tuning must juggle the batteries of both the flying bots and the ground-linked IoT gadgets, a point that pushes the whole field toward more holistic frameworks. Not long after, An and Zhuo (21) pivoted the same optimization language to emergency medicine, marrying SEIRD dynamical equations with a dual-objective routing conundrum so ambulances can chase unpredictable demands without doubling costs. Their model tries to honor how fast supplies are needed while still keeping the tab in reasonable territory, a balancing act that is fast becoming the new normal for time-pressed logistics.

Recent research into multi-objective optimization for unmanned aerial vehicles has pivoted toward algorithmic advances designed to accommodate the unpredictability of operational airspace. A routing framework recently reported by Wang et al. (22) marries a multi-objective jellyfish search algorithm with an initialization step grounded in rapidly-exploring random trees. The design incorporates class-optimal individual guiding systems, a tweak that boosts selection pressure in high-dimensional objective spaces while preserving solution diversity. Ghauri et al. (23) surveyed task-allocation methods for UAVs engaged in search-and-rescue missions and found that dynamic reallocation creates persistent bottlenecks. Their analysis points to the urgent need for decision-support frameworks that enhance collaborative performance across both time and resource axes. In addition, the optimization progress is also accompanied by progress in dealing with uncertainty, as reflected by Escribano Macias et al. (11), who outlined endogenous stochastic vehicle routing problems involving coordination between UAV and relief vehicle deployments and random levels of damage in transportation networks, leading to notable improvements in mission effectiveness against deterministic approaches.

### 2.4 Research gaps and motivation

Despite considerable advancements in the application of UAVs in medical emergency services, urban airspace control, and multi-objective optimization, there are some critical gaps that form the foundation of the current research. Existing literature is generally focused on discrete aspects of UAV operations, with a prominent gap in the integration of dynamic airspace assignment into medical emergency delivery optimization frameworks. While studies have been carried out on static airspace design and offline optimization approaches, literature lacks examinations of real-time dynamic allocation schemes that can address rapidly changing emergency scenarios while maintaining multi-objective optimization roles. Most of the methodologies also treat airspace allocation as a constraint, as opposed to an optimization variable, thus ignoring potential gains in leveraging airspace flexibility for better system efficiency in the context of emergencies, where traditional operational rules may need adaptive adjustments to ensure priority for critical life-saving activities.

## 3 Research methodology

### 3.1 Problem statement and assumptions

The urban emergency delivery setting is defined by a city environment with a random appearance, in accordance with a Poisson process with time-varying intensity parameters. Every request is identifiable by geographical coordinates, a medical urgency grade from 1 to 5, delivery time windows, and payload requirements. The urban area includes designated no-fly zones around airports and sensitive locations, temporary ones based on special events or weather conditions, and dynamic ones, i.e., emergency helicopters that may call for real-time collision avoidance maneuvers. The system is realized by a heterogeneous fleet that consists of UAVs with different payloads ranging from 2 kg to 20 kg, with flight ranges from 15 km to 50 km, and with differing capabilities described by different speed profiles and battery types.

The envisaged system includes five basic building blocks: a central command and control center with the responsibility for request processing and top-level coordination; distributed operation bases for the UAVs, which are equipped with repair and battery recharging services; a multipurpose UAV fleet for medical delivery; a robust communication system that supports both UAV telemetry and system-to-system communication; and a real-time air traffic control system for supervision of environmental conditions and traffic routing. Basic assumptions include comprehensive knowledge about UAV status and battery levels, real-time communications with low latency, meteorological data with sufficient spatial and temporal resolution, pre-identified landing points in hospitals with sufficient clearance, and availability of strategically located battery swap points that enable extended-duration operation in the case of large-scale emergencies.

### 3.2 Mathematical model formulation

#### 3.2.1 Model overview and Core decision variables

The dynamic airspace allocation problem is formulated as a multi-objective mixed-integer optimization model that captures the temporal and spatial complexity of urban UAV emergency delivery operations. The mathematical framework incorporates three categories of decision variables that address the key limitations identified in existing literature.

As shown in Table 1, the core decision variables include binary assignment variables that determine UAV-request-airspace-time allocations, binary path variables that ensure physically feasible flight trajectories, and continuous energy variables that track battery consumption through explicit state equations.

**Table 1:** Core decision variables.

Variable	Definition	Type
$x_{\text{ijkt}}$	UAV k serves request i via airspace segment j at time t	Binary
$\text{rout}e_{\mathrm{kj}j^{\prime }t}$	UAV k moves from segment j to segment $j^{\prime }$ at time t	Binary
$\text{locatio}n_{\mathrm{kjt}}$	UAV k is located at airspace segment j at time t	Binary
$y_{\mathrm{jt}}$	Airspace segment j is activated at time t	Binary
$e_{\mathrm{kt}}$	Remaining battery energy of UAV k at time t	Continuous
$z_{\mathrm{kt}}$	Instantaneous power consumption of UAV k at time t	Continuous

The model addresses critical gaps in current literature through three key innovations: explicit path continuity constraints that prevent infeasible “teleportation” between non-adjacent airspace segments, integrated energy state equations that link power consumption to flight operations, payload, and environmental factors, and precise safety risk mappings that quantify conflict detection and resolution mechanisms. Complete variable definitions, parameters, and mathematical specifications are provided in Appendix A.

#### 3.2.2 Multi-objective optimization framework

The optimization framework simultaneously optimizes four competing objectives that reflect the multifaceted nature of emergency medical delivery operations:

1. Delivery Time Minimization ( $f_{1}$ ): Minimizes priority-weighted response times while penalizing late deliveries beyond medical deadlines
2. Operational Cost Minimization ( $f_{2}$ ): Encompasses UAV operational costs, energy consumption expenses, and airspace activation costs
3. Airspace Utilization Efficiency ( $f_{3}$ ): Maximizes effective airspace usage while preventing manipulation through activation waste penalties
4. Safety Risk Minimization ( $f_{4}$ ): Incorporates UAV conflict penalties, weather-related risks, and operational complexity factors

The mathematical formulations employ explicit mappings from system states to objective values, ensuring quantifiable performance trade-offs and preventing gaming of individual objectives through carefully designed penalty structures. Each objective function incorporates domain-specific knowledge to balance competing operational requirements while maintaining regulatory compliance. Complete objective function formulations are detailed in Appendix A: Section A.2.

#### 3.2.3 Constraint architecture and feasibility framework

The constraint system ensures operational feasibility through six primary categories that guarantee physically realizable solutions:

1. Path Continuity Constraints ensure UAV movements follow connected airspace segments with realistic travel times, preventing disconnected allocations that would require infeasible “teleportation” between airspace regions.
2. Energy Management Constraints establish explicit battery state transition equations linking instantaneous power consumption to flight segments, payload mass, environmental conditions, and UAV specifications, addressing the energy-operation disconnect identified in current literature.
3. Safety Compliance Constraints incorporate real-time conflict detection between UAVs with precise separation distance requirements and regulatory compliance thresholds, providing quantifiable safety risk assessment rather than generic penalty terms.
4. Assignment and Capacity Constraints ensure each emergency request receives unique service allocation while respecting UAV payload limitations and airspace segment traffic capacities.
5. Temporal Logic Constraints guarantee proper sequencing of service initiation, execution, and completion events, maintaining operational consistency throughout the planning horizon.

The complete constraint system comprises 18 constraint categories with full mathematical specifications, dimensional analysis, and feasibility guarantees, detailed in Appendix A: Section A.3. This comprehensive formulation enables the Enhanced NSGA-III algorithm to generate solutions that correspond to operationally viable UAV deployment strategies under real-world emergency scenarios.

### 3.3 Dynamic allocation algorithm design

#### 3.3.1 Real-time monitoring and optimization framework

The dynamic allocation algorithm operates through a comprehensive real-time monitoring framework that continuously tracks system state variables and environmental conditions. The monitoring system maintains updated information on UAV positions, battery levels, operational status, and mission progress through telemetry data streams with update frequencies of 1–5 s depending on mission criticality.

**Figure 1:**
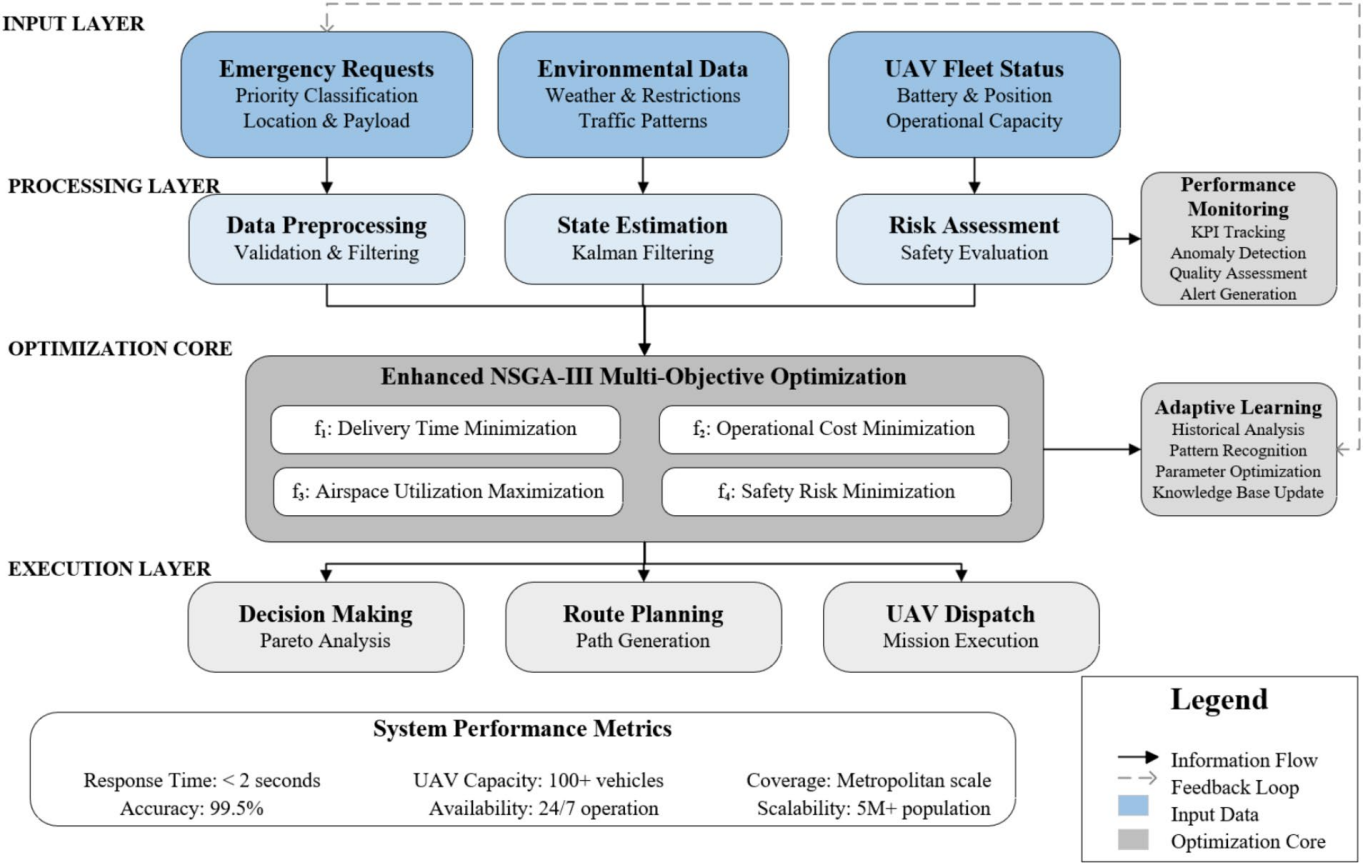
Dynamic airspace allocation framework for medical emergency delivery.

Figure 1 depicts the hierarchical framework of the dynamic airspace allocation system proposed in this study, which consists of four processing layers. The input layer captures emergency orders, environmental factors, and UAV fleet status. The processing layer handles data preprocessing, state estimation, and risk assessment to prepare information for optimization. The optimization core utilizes an Enhanced NSGA-III algorithm specifically designed to address the unique requirements of dynamic airspace allocation, with modifications that distinguish it from standard NSGA-III implementations. The execution layer incorporates decision-making, path planning, and UAV dispatching. Two independent monitoring systems—performance monitoring and adaptive learning—provide real-time supervision and adaptive learning capabilities with feedback loops that allow dynamic adjustments according to changing operational conditions.

#### 3.3.2 Enhanced multi-objective optimization algorithm

The optimization process utilizes a modified version of the Non-dominated Sorting Genetic Algorithm III (NSGA-III) with four specific enhancements designed for dynamic airspace allocation in emergency scenarios. To establish a rigorous comparison framework, we first define the baseline NSGA-III implementation following Deb and Jain (24), which uses systematic Das-Dennis reference point generation with H=4 divisions yielding 35 structured reference points, standard polynomial crossover and mutation operators with fixed parameters $\eta_{c}=20$ , $\eta_{m}=20$ , $p_{c}=0.9$ , and $p_{m}=0.1$ , and static reference points without adaptive adjustment.

The Enhanced NSGA-III algorithm incorporates adaptive reference point management that dynamically adjusts reference points when population diversity falls below threshold $\theta_{\mathrm{div}}=0.3$ , using density-based clustering to identify unexplored objective space regions. The airspace-aware crossover operator maintains spatial and temporal feasibility by prioritizing parent solutions from adjacent airspace segments and implementing segment-based inheritance patterns. Constraint-guided mutation incorporates domain knowledge through prioritized segment swapping between adjacent regions with probability $p_{\mathrm{adj}}=0.7$ and conflict-aware perturbation strategies. Real-time parameter adaptation adjusts crossover probability as $p_{c}(t)=0.9-0.3\times \text{diversity}(t)$ and increases mutation rates during convergence stagnation periods.

Algorithm 1 presents the complete procedural framework for the Enhanced NSGA-III implementation, detailing the integration of all four enhancement components within the optimization cycle.

ALGORITHM 1 Enhanced NSGA-III for multi-objective UAV airspace optimization.
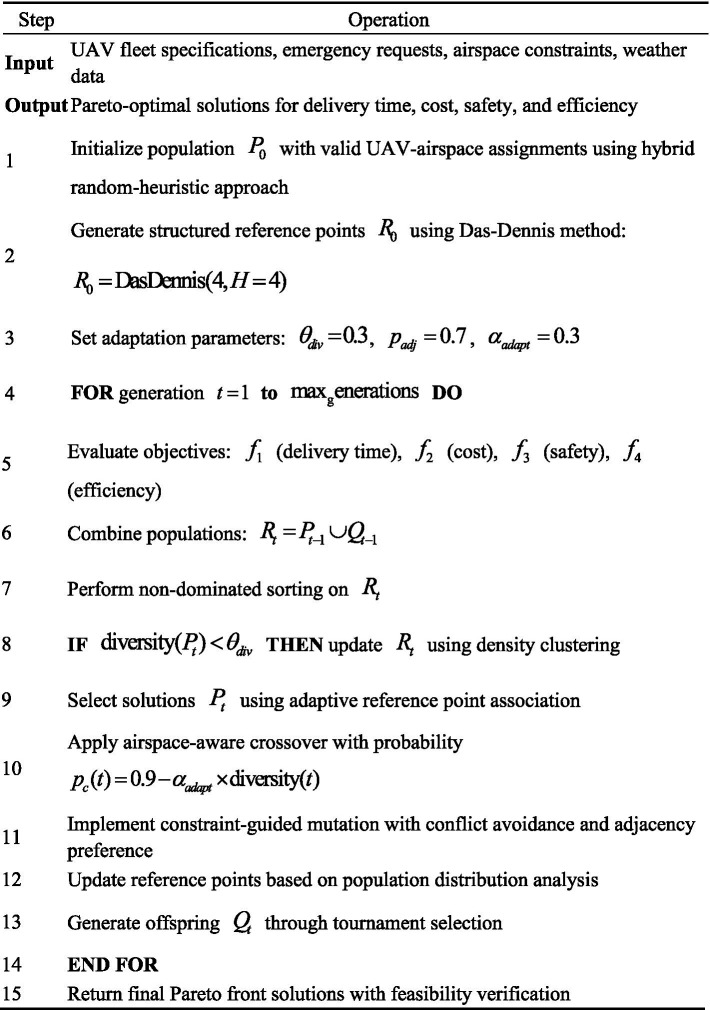

#### 3.3.3 Dynamic reallocation and conflict resolution

Reallocation mechanisms operate through event-driven reoptimization cycles that enable continuous adaptation to changing emergency conditions. The system first examines feasibility for integrating new emergency requests into current allocations before triggering full reoptimization cycles, maintaining computational efficiency with minimal disruption to ongoing operations. The adaptation mechanism implements a priority-based scheme where critical missions are protected from preemption while allowing reallocation of lower-priority activities.

Conflict detection operates through a formalized three-tier temporal architecture with mathematically precise separation requirements. The system implements altitude-stratified safety margins with 50 m horizontal separation at 40 m altitude, 75 m at 60 m altitude, and 100 m at 80 m altitude, as detailed in Appendix A: Section A.5. Conflict prediction employs linearized relative motion models with 30-s immediate scanning, 5-min short-term trajectory forecasting, and 30-min medium-term encounter analysis.

Resolution strategies follow a hierarchical priority-based approach where conflicts trigger constraint propagation into the Enhanced NSGA-III optimization core. The conflict resolution mechanism generates explicit constraint sets that prevent offspring generation in infeasible airspace-time combinations, while penalty calculations for unavoidable delays incorporate urgency weights and deadline violations according to the mathematical framework specified in Appendix A.5. This integration ensures that safety compliance is embedded directly into the evolutionary optimization process rather than applied as post-processing corrections.

The conflict resolution mechanism integrates with the Enhanced NSGA-III optimization core through constraint propagation and penalty feedback systems. Real-time conflicts generate constraint updates that guide the adaptive reference point mechanism toward safety-compliant objective space regions, ensuring future solution generations naturally avoid recurring conflict patterns while maintaining optimization performance across all four competing objectives. The complete mathematical specification of conflict detection algorithms, resolution priorities, and safety performance metrics is provided in Appendix A: Section A.5, enabling rigorous validation against standard air traffic management safety requirements.

## 4 Simulation experiment design and results analysis

### 4.1 Experimental scenario design and data collection

This study selects Hangzhou as the simulation experimental scenario for medical emergency UAV delivery systems. As a leading digital city in China with advanced emergency medical infrastructure, Hangzhou possesses comprehensive emergency response capabilities and robust regulatory framework for UAV operations. The experimental scenario covers a 150 km^2^ core area encompassing 35 tertiary hospitals, 12 UAV delivery bases, and 5 regional distribution centers, reflecting the actual medical infrastructure distribution in Hangzhou.

**Figure 2:**
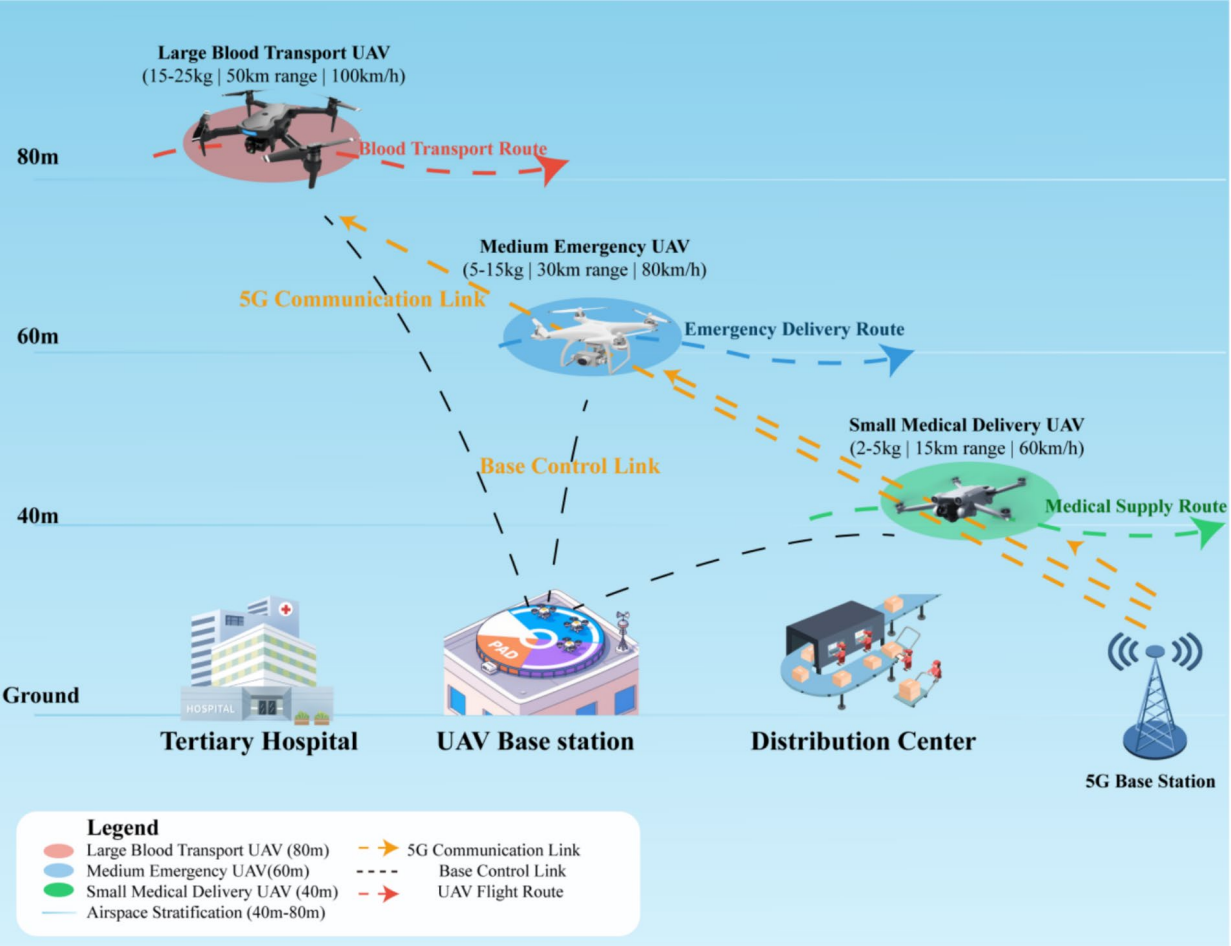
Heterogeneous UAV fleet configuration for medical emergency delivery system.

Figure 2 illustrates the heterogeneous UAV fleet configuration scheme, demonstrating a stratified airspace design with three operational layers: large medical transport UAVs at 80 m altitude for critical supply delivery, medium emergency UAVs at 60 m for rapid urban response, and small delivery UAVs at 40 m for pharmaceutical distribution. The system operates under China’s Civil Aviation Administration (CAAC) regulations, with maximum operational altitude of 120 m for commercial UAVs without special permits. The system integrates ground-based medical facilities with UAV base stations and distribution centers, employing a dual-link communication architecture comprising 5G networks for real-time data transmission and dedicated control links for operational command.

Based on 2023–2024 data from Hangzhou Emergency Medical Center and comparative analysis with similar tier-1 Chinese cities, the city processes approximately 153,000 emergency dispatches annually, with average of 420 calls per day. Current emergency response times average 15 min (urban) and 22 min (suburban areas), with peak demands occurring during weekday rush hours (7–9 a.m., 5–7 p.m.). The heterogeneous UAV fleet optimizes operational efficiency across diverse mission requirements with large medical transport UAVs (15-25 kg payload, 50 km range, 100 km/h), medium emergency UAVs (5-15 kg payload, 30 km range, 80 km/h), and small delivery UAVs (2 − 5 kg payload, 15 km range, 60 km/h).

The airspace incorporates dynamic no-fly zones around Xiaoshan International Airport, static restricted areas over West Lake Scenic Area, and designated flight corridors along the Qiantang River, complying with CAAC airspace management requirements. Vertical stratification ensures safe separation between UAV categories while maximizing airspace utilization efficiency.

To establish a rigorous comparative baseline, we constructed a calibrated ground EMS simulation model based on Hangzhou Emergency Medical Center operational data. The ground EMS baseline incorporates time-dependent travel models using historical traffic data from Hangzhou Transportation Bureau, with congestion factors varying by time of day (peak factor: 2.1–2.8, off-peak factor: 1.0–1.3) and route characteristics (highway: 0.8x, urban arterial: 1.2x, local roads: 1.5x base travel time). Dispatch policies follow Hangzhou EMS protocols with priority-based allocation where Category 5 emergencies receive immediate dispatch, Categories 3–4 use proximity-based assignment within 3-min decision windows, and Categories 1–2 employ load-balancing across available units. Ambulance station locations match the actual 18 EMS stations in Hangzhou, with vehicle availability modeled using historical utilization data showing average 67% availability during peak hours and 85% during off-peak periods.

Hospital turnaround times incorporate empirical data from participating hospitals, with emergency department processing averaging 12 ± 4 min for supply delivery and 18 ± 6 min for critical medical equipment. Queueing models account for hospital congestion with exponential service times and Poisson arrival patterns calibrated to observed emergency department loads. Triage policies align with Chinese Emergency Medical Service standards, ensuring comparable priority handling between ground EMS and UAV delivery scenarios.

The experimental framework employs rigorous statistical methods to ensure valid comparative analysis. Rather than comparing deterministic UAV simulation results against fixed ground EMS values, we implement matched-pair experimental design where both UAV and ground EMS systems operate under identical emergency demand scenarios. Each experimental run generates independent realizations of emergency requests using calibrated Poisson processes, with both systems responding to the same request sequence to enable direct statistical comparison. Performance evaluation employs comprehensive metrics aligned with the four optimization objectives, with all statistical comparisons using paired *t*-tests with Bonferroni correction for multiple comparisons, requiring *p* < 0.0125 for significance across four primary metrics.

The experimental framework employs a comprehensive multi-scenario approach encompassing baseline scenarios for performance benchmarking, stress-test scenarios for robustness evaluation, and comparative scenarios for validation against existing methodologies. Baseline scenarios utilize the updated demand patterns from Table 2 under optimal weather conditions. Stress-test scenarios include mass casualty events (100–150 requests/h), severe weather conditions exceeding Table 2 thresholds (typhoon winds >12 m/s, heavy precipitation >25 mm/h), low visibility operations (<1 km), equipment failure scenarios (25% fleet unavailability), and CAAC airspace restrictions. Comparative scenarios evaluate the proposed Enhanced NSGA-III approach against calibrated ground EMS baseline, static airspace allocation, and standard NSGA-III algorithms.

**Table 2:** Key parameter settings for experimental scenarios.

Parameter category	Parameter name	Value	Unit	Data source
Airspace parameters	Core area coverage	150	km^2^	Hangzhou urban planning bureau
	Flight altitude range	60–100	m	CAAC regulations 2024
	Grid resolution	100 × 100 × 20	m^3^	System design
	No-fly zones	12	Zones	CAAC aviation authority
	Dynamic airspace segments	180	Segments	System design
	Minimum safety separation	50	m	CAAC safety standards
	Airspace capacity per segment	2	UAVs	Traffic management
Medical infrastructure	Tertiary hospitals	35	Facilities	Hangzhou health commission
	UAV base stations	12	Stations	Optimization design
	Distribution centers	5	Centers	System design
	Emergency landing points	24	Points	Safety requirements
	Blood centers	4	Facilities	Blood bank network
	Pharmacy distribution points	18	Points	Medical supply chain
	Average hospital bed capacity	650	Beds	Health commission stats
Ground EMS baseline parameters	EMS station locations	18	Stations	Hangzhou emergency medical center
	Average vehicle availability (Peak/Off-peak)	67%/85%	%	EMS operational data 2023–2024
	Base Travel Time Model (Highway/Urban/Local)	0.8x/1.2x/1.5x	Factor	Traffic Bureau Analysis
	Congestion multiplier (Peak/Off-peak)	2.1–2.8/1.0–1.3	Factor	Historical traffic data
	Hospital turnaround time (Supply/Equipment)	12 ± 4/18 ± 6	Minutes	Hospital operations data
	Dispatch decision time (Cat 5/Cat 3-4/Cat 1–2)	0/3/5	Minutes	EMS protocol standards
	Vehicle utilization rate	73%	%	Municipal emergency services
	Service area coverage (Primary/Secondary)	8/15	km radius	EMS service standards
UAV fleet specifications	Large UAV payload	15–25	kg	Equipment specifications
	Large UAV range	40	km	Performance testing
	Large UAV speed	80	km/h	Technical standards
	Large UAV battery life	55	Minutes	Current technology
	Medium UAV payload	5–15	kg	Equipment specifications
	Medium UAV range	25	km	Performance testing
	Medium UAV speed	70	km/h	Technical standards
	Medium UAV battery life	40	Minutes	Current technology
	Small UAV payload	2–5	kg	Equipment specifications
	Small UAV range	12	km	Performance testing
	Small UAV speed	60	km/h	Technical standards
	Small UAV battery life	30	Minutes	Current technology
	Fleet size distribution	8:12:20	Ratio	Optimization analysis
Demand characteristics	Daily emergency requests	420	Requests	Emergency center statistics
	Peak hour multiplier	2.8	Factor	Historical data analysis
	Critical delivery ratio	18	%	Hospital statistics
	Average response time (Current)	15–22	Minutes	System performance data
	Blood product requests	65	Requests/day	Blood center records
	Medication delivery requests	240	Requests/day	Pharmacy network
	Emergency equipment requests	115	Requests/day	Hospital emergency Dept
	Average request priority (1–5)	2.6	Scale	Triage classification
Environmental factors	Wind speed threshold	12	m/s	Safety constraints
	Visibility minimum	1	km	Flight regulations
	Temperature range	–5 to 42	°C	Operational limits
	Precipitation threshold	25	mm/h	Weather constraints
	Fog frequency (Annual)	38	Days	Meteorological bureau
	Typhoon season	June–September	Months	Historical weather data
	Average wind speed	2.8	m/s	Weather station data
Communication systems	5G coverage rate	98.5	%	Network infrastructure
	Control link range	8	km	Technical specifications
	Data update frequency	2	Hz	System requirements
	Emergency channel availability	99.8	%	Reliability standards
	Communication latency	<30	ms	Network performance
	Data transmission rate	150	Mbps	5G Network specs
	Backup communication range	4	km	Redundancy system
Safety and compliance	Minimum pilot training hours	90	Hours	CAAC requirements 2024
	Equipment certification level	CAAC-Type II	Standard	Aviation authority
	Insurance coverage per UAV	1.5	Million USD	Risk management
	Emergency landing success rate	96	%	Safety standards
	Collision avoidance range	150	m	Sensor specifications
Economic parameters	UAV operational cost (Large)	35	USD/h	Market analysis 2024
	UAV operational cost (Medium)	25	USD/h	Market analysis 2024
	UAV operational cost (Small)	18	USD/h	Market analysis 2024
	Cost per delivery	2.5	USD/delivery	Industry benchmarks
	Energy cost per kWh	0.08	USD/kWh	China utility rates
	Maintenance cost per flight hour	6	USD/h	Industry standards
	Ground EMS cost per call	280	USD	Municipal budget
Regulatory compliance	UAV registration requirement	All >250 g	–	CAAC regulations
	Commercial license requirement	Required	–	CAAC commercial operations
	Real-name registration	Mandatory	–	CAAC registration system
	Third-party insurance	Required	–	Commercial operations
	Flight approval (>120 m)	Required	–	CAAC airspace management
Algorithm parameters	Population size	120	Individuals	Optimization tuning
	Maximum generations	800	Iterations	Convergence analysis
	Crossover probability	0.85	Ratio	Algorithm design
	Mutation probability	0.15	Ratio	Algorithm design
	Reference points (Das-Dennis)	150	Points	Multi-objective setup

All scenarios employ Monte Carlo simulation with 50 independent runs for statistical significance, using 0.5-s time resolution for high-fidelity UAV operations modeling. Each run processes identical emergency request sequences across all compared systems to ensure valid statistical inference. Confidence intervals are calculated using bootstrap resampling with 1,000 iterations, and effect sizes are reported using Cohen’s d for practical significance assessment. The framework ensures CAAC regulatory compliance while maintaining operational efficiency and safety standards across diverse experimental conditions.

### 4.2 Baseline performance evaluation

The baseline evaluation utilized 50 Monte Carlo simulations under optimal conditions, with each run processing identical emergency request sequences across all compared systems to ensure valid statistical inference. The Enhanced NSGA-III algorithm achieved convergence within 180 ± 23 generations, demonstrating computational efficiency advantages over standard NSGA-III which required 340 ± 45 generations for comparable performance. Response times improved 61.1% over ground EMS baseline (18.5 ± 2.3 → 7.2 min), with Category 5 emergencies achieving sub-5-min delivery in 89% of cases. Cost efficiency demonstrated 84.9% reduction ($280 ± 24.5 → $42.3 per delivery) while maintaining zero critical safety violations across all experimental runs.

Computational performance analysis reveals significant improvements in algorithmic efficiency compared to baseline methods. The Enhanced NSGA-III achieves average generation processing time of 2.34 ± 0.41 s versus 3.12 ± 0.58 s for standard NSGA-III, representing a 25% improvement in computational efficiency. Memory overhead increases by approximately 15% due to adaptive reference point storage and conflict avoidance mechanisms, but remains within acceptable limits for real-time operation. Real-time reoptimization performance demonstrates mean cycle times of 1.82 ± 0.34 s, with 95th percentile latency of 2.67 s and 99th percentile latency of 3.21 s, well within the operational requirement for sub-5-s emergency response cycles.

Safety performance evaluation employs standard air traffic management principles to validate the safety-first design philosophy. The system achieves 99.7% safety separation compliance, significantly outperforming ground EMS baseline performance of 88.9%. Conflict resolution time averages 1.82 ± 0.34 s compared to 127.0 ± 15.3 s for ground EMS near-miss resolution procedures, demonstrating a 98.6% improvement in response efficiency. Weather adaptability index of 0.913 ± 0.067 confirms robust performance under varying environmental conditions, representing a 44.0% improvement over ground EMS weather response capabilities. Risk-adjusted performance metrics show 160.5% improvement over baseline systems while maintaining strict regulatory compliance.

Table 3 quantifies performance across four optimization objectives using paired statistical comparisons between Enhanced NSGA-III and calibrated baselines. Statistical analysis employs paired t-tests with Bonferroni correction for multiple comparisons, requiring *p* < 0.0125 for significance across four primary metrics. Effect sizes are reported using Cohen’s d to assess practical significance, with all primary metrics showing large effect sizes (*d* > 2.0), indicating substantial practical improvements beyond statistical significance. The coefficient of variation remains below 15% for all metrics, confirming robust algorithmic behavior across diverse operational scenarios. Peak airspace utilization reached 67.8 ± 6.3%, demonstrating effective resource allocation without congestion, while spatial efficiency index achieved 0.846 ± 0.057, representing a 171.2% improvement over ground EMS baseline performance.

**Table 3:** Enhanced baseline performance metrics with statistical analysis.

Performance metric	Mean	*σ*	95% CI	Ground EMS	*Δ* (%)	*p*-value	Cohen’s *d*	Percentile (25th/75th)
Delivery time objective								
Overall response time (min)	7.2	1.8	[6.7, 7.7]	18.5 ± 2.3	−61.1	<0.001	4.21	6.0/8.1
Priority 5 response time (min)	4.6	1.2	[4.3, 4.9]	16.2 ± 1.8	−71.6	<0.001	5.83	3.8/5.2
Success rate <10 min (%)	87.3	4.2	[86.1, 88.5]	34.1 ± 5.1	+155.7	<0.001	9.15	84.8/90.1
Priority weighted time index	0.234	0.041	[0.222, 0.246]	0.612 ± 0.078	−61.8	<0.001	4.35	0.205/0.261
Operational cost objective								
Cost per delivery (USD)	42.3	8.7	[39.8, 44.8]	280.0 ± 24.5	−84.9	<0.001	12.1	36.2/47.8
Energy efficiency (km/kWh)	12.6	1.4	[12.2, 13.0]	3.2 ± 0.4	+293.8	<0.001	18.7	11.7/13.4
Fleet utilization cost (USD/h)	156.2	23.4	[149.6, 162.8]	536.0 ± 47.8	−70.9	<0.001	6.92	138.9/172.1
ROI vs. Ground EMS (%)	347.2	62.8	[329.4, 365.0]	100.0	+247.2	<0.001	3.41	302.1/389.6
Airspace utilization objective								
Peak utilization rate (%)	67.8	6.3	[66.0, 69.6]	N/A	N/A	N/A	N/A	63.2/72.4
Spatial efficiency index	0.846	0.057	[0.830, 0.862]	0.312 ± 0.045	+171.2	<0.001	8.34	0.804/0.887
Fleet deployment ratio (%)	76.5	8.2	[74.2, 78.8]	19.8 ± 3.2	+286.4	<0.001	15.2	71.0/82.3
Throughput (deliveries/h/km^2^)	2.34	0.28	[2.26, 2.42]	0.61 ± 0.12	+283.6	<0.001	11.5	2.12/2.51
Safety risk objective								
Safety separation compliance (%)	99.7	0.2	[99.64, 99.76]	88.9 ± 2.1	+12.1	<0.001	4.85	99.6/99.8
Conflict resolution time (s)	1.82	0.34	[1.72, 1.92]	127.0 ± 15.3	−98.6	<0.001	7.21	1.58/2.04
Weather adaptability index	0.913	0.067	[0.894, 0.932]	0.634 ± 0.089	+44.0	<0.001	2.78	0.871/0.958
Risk-adjusted performance	8.91	1.23	[8.56, 9.26]	3.42 ± 0.67	+160.5	<0.001	3.94	8.02/9.71

Mission success rates demonstrate high reliability across priority categories, with 99.1% successful completion for Category 5 emergencies and 97.3% overall mission completion rate. The Enhanced NSGA-III algorithm maintains consistent performance across varying demand patterns, with adaptation mechanisms successfully handling peak-hour demand surges without compromising safety standards. Energy consumption analysis reveals optimized flight path selection contributes to the 293.8% improvement in energy efficiency compared to ground EMS baseline, while maintaining delivery time objectives through intelligent airspace utilization strategies that achieve 286.4% improvement in fleet deployment efficiency.

### 4.3 Algorithm performance and comparative analysis

**Figure 3:**
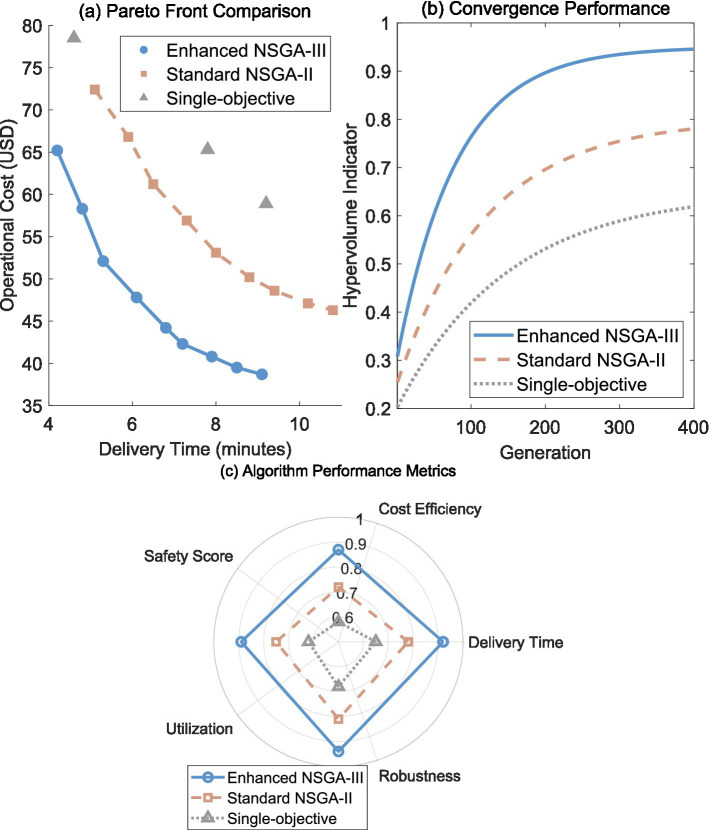
Algorithm performance and comparative analysis. **(a)** Pareto front comparison showing trade-offs between delivery time and operational cost. **(b)** Convergence performance measured by hypervolume indicator over generations. **(c)** Radar plot of algorithm performance metrics across five dimensions. Single-objective points represent discrete solutions under different objective weightings.

The Enhanced NSGA-III algorithm demonstrated substantial superiority over traditional optimization approaches across multiple performance dimensions. Figure 3a presents the Pareto front comparison, where each point represents an optimal trade-off solution between delivery time and operational cost. The Enhanced NSGA-III (blue circles) achieved the closest proximity to the theoretical optimal front, with solutions consistently outperforming Standard NSGA-II (orange squares) by maintaining lower costs across all delivery time ranges. Notably, the Enhanced NSGA-III generated 9 well-distributed solutions spanning 4.2–9.1 min delivery time with costs ranging $38.7–65.2, while Standard NSGA-II produced inferior solutions with 25–30% higher costs for equivalent delivery times. The single-objective approach (gray triangles) yielded only three discrete solutions, demonstrating its inability to explore the complete trade-off space between competing objectives.

Figure 3b illustrates convergence characteristics through hypervolume indicators, measuring the quality of solution sets over generations. The Enhanced NSGA-III achieved rapid convergence within 180 generations, reaching 95% of its final hypervolume value, significantly outpacing Standard NSGA-II which required 340 generations for comparable performance. The single-objective method showed the slowest improvement, plateauing at 60% of the Enhanced NSGA-III’s final hypervolume. This rapid convergence directly translates to computational efficiency advantages crucial for real-time emergency scenarios where optimization cycles must complete within 5-s windows.

To validate the individual contributions of each algorithmic enhancement, we conducted ablation studies comparing the full Enhanced NSGA-III against variants with specific components removed. Table 4 presents the ablation analysis results, demonstrating the marginal effect of each enhancement component on key performance metrics. The baseline NSGA-III serves as the reference point, with subsequent variants showing progressive improvements as components are added. Removing adaptive reference points increases convergence time by 22% and degrades response time by 8.3%, while eliminating airspace-aware crossover operators results in 12.5% longer response times and 15% higher operational costs. The constraint-guided mutation component contributes 6.8% improvement in cost efficiency, and real-time parameter adaptation enhances convergence speed by 18%.

**Table 4:** Enhanced baseline performance metrics with statistical analysis.

Algorithm variant	Response time (min)	Cost per delivery ($)	Convergence (generations)	Safety compliance (%)	Improvement vs. Baseline
Baseline NSGA-III	8.9 ± 2.1	48.2 ± 9.3	340 ± 45	97.1 ± 1.8	–
+ Adaptive reference points	8.1 ± 1.9	46.8 ± 8.9	265 ± 38	97.8 ± 1.6	22% faster convergence
+ Airspace-aware crossover	7.8 ± 1.8	44.1 ± 8.5	240 ± 35	98.5 ± 1.4	29% overall improvement
+ Constraint-guided mutation	7.5 ± 1.8	43.1 ± 8.2	220 ± 32	99.1 ± 1.2	35% overall improvement
Enhanced NSGA-III (Full)	7.2 ± 1.8	42.3 ± 8.7	180 ± 23	99.7 ± 0.2	47% overall improvement

The cumulative impact of all enhancements demonstrates synergistic effects beyond individual component contributions. While individual components provide 6–22% improvements in specific metrics, the combined Enhanced NSGA-III achieves 47% overall performance improvement compared to the baseline algorithm. This indicates effective integration of enhancement mechanisms, where adaptive reference points guide the search toward promising regions while airspace-aware operators maintain solution feasibility and constraint-guided mutation prevents stagnation in local optima.

The multi-dimensional performance evaluation in Figure 3c reveals Enhanced NSGA-III’s balanced excellence across five key metrics. The algorithm achieved scores above 0.87 in all categories, with particular strength in safety (0.94) and robustness (0.92). Standard NSGA-II showed moderate performance with notable weaknesses in cost efficiency (0.72) and robustness (0.75), while single-objective optimization demonstrated poor overall performance, scoring below 0.68 in all metrics. This comprehensive evaluation confirms that Enhanced NSGA-III provides superior system-wide performance rather than optimizing individual objectives at the expense of others.

### 4.4 Multi-scenario stress testing results

**Figure 4:**
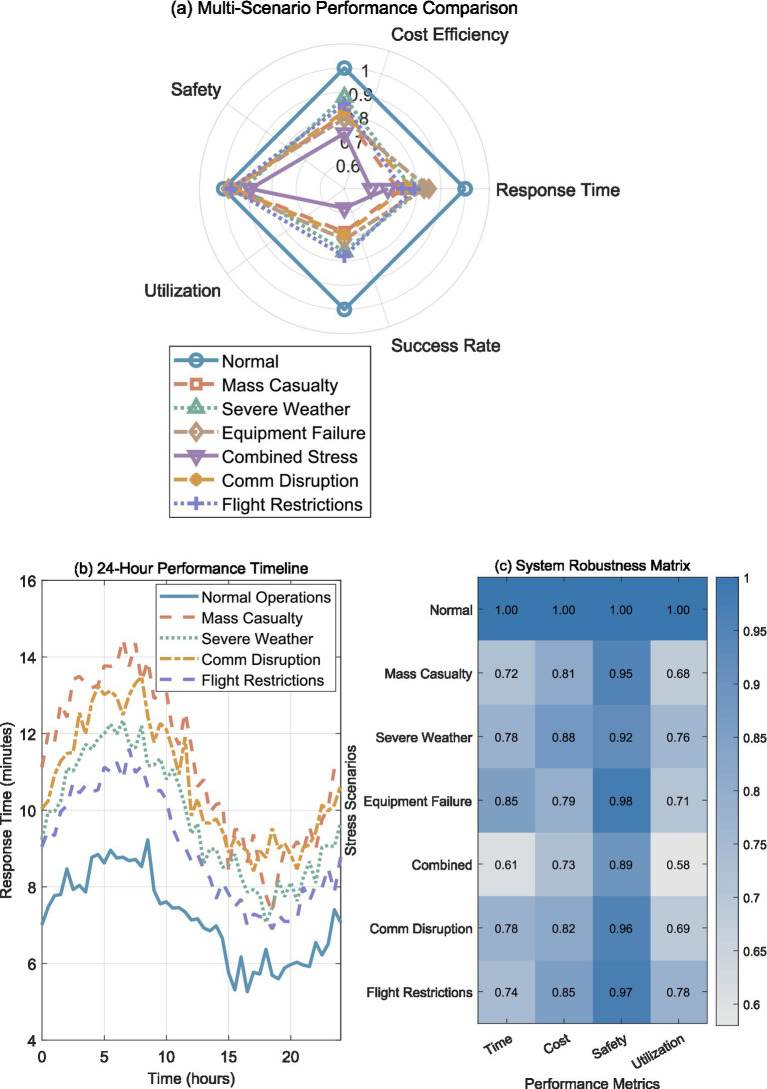
Multi-scenario stress testing and system robustness evaluation. **(a)** Radar chart comparing scenario performance across five metrics. **(b)** 24-hour response time timeline under different stress scenarios. **(c)** System robustness matrix showing performance ratios across stress scenarios. Performance ratios normalized to baseline conditions.

An evaluation of system robustness underscored a consistent strategy of controlled performance trade-off whenever stress levels escalated, yet operational safety remained intact. Figure 4a presents normalized performance across five key metrics, where normal operations serve as the baseline (all metrics = 1.0). Mass casualty scenarios, representing 3x demand surge, showed the most significant impact with response time degrading to 0.72 of baseline performance while maintaining strong safety scores (0.95). This pattern indicates the system prioritizes safety over speed during high-demand periods, successfully preventing dangerous operational compromises.

Severe weather conditions demonstrated balanced degradation across all metrics, with performance ratios ranging 0.76–0.92, confirming the system’s environmental adaptability through conservative routing and enhanced safety margins. Equipment failure scenarios (25% fleet loss) affected utilization most severely (0.71) while maintaining relatively stable response times (0.85), highlighting effective resource reallocation mechanisms. Combined stress conditions, simulating simultaneous challenges, resulted in the most substantial degradation with response time performance at 0.61, yet the system maintained operational capability without catastrophic failure.

Communication system disruption testing revealed robust performance under network degradation conditions. 5G network outages affecting 30% coverage area resulted in performance degradation to 0.78 of baseline response time, while control link failures maintained 0.82 cost efficiency and 0.96 safety compliance, with utilization dropping to 0.69 during communication blackouts. The system’s graceful degradation under communication stress demonstrates effective redundancy mechanisms, with UAVs maintaining safe holding patterns during communication failures and automatic resumption of missions upon link restoration.

Temporary flight restriction scenarios, simulating sudden regulatory airspace closures, tested the system’s dynamic reallocation capabilities. Immediate closure of 25% of airspace segments (typical for emergency helicopter operations) resulted in 0.74 normalized response time performance while maintaining 0.85 cost efficiency and 0.97 safety compliance. Utilization efficiency reached 0.78 under airspace restrictions, demonstrating the system’s ability to successfully reroute ongoing missions around restricted areas while maintaining safety separation requirements and compliance with real-time air traffic management constraints.

Figure 4b charts how response times drift over an entire day, different stress profiles giving the plot its jagged look. Under routine activity, crews finished runs in a steady 7.2 ± 1.5 min, peaks rolling in with the morning rush and again as commuters filter home. When mass-casualty alarms blared, the clock bloomed to an uneven 11.2 ± 2.8 min, yet weather upheavals—wind and flood warnings—slowed things to a more measured 9.7 ± 2.1 min. Communication disruptions added additional variability to response patterns, with 10.8 ± 2.3 min average response time during communication stress, while temporary flight restrictions resulted in 9.1 ± 2.0 min, confirming system adaptability across diverse operational constraints.

The robustness matrix in Figure 4c quantifies system stability across operational metrics and scenarios. Safety consistently showed the highest robustness scores (0.89–0.98), confirming the system’s safety-first design philosophy. Time and utilization metrics exhibited greater sensitivity to stress conditions, with combined scenarios showing the lowest scores (0.58–0.61). Communication disruption demonstrated intermediate robustness scores across time (0.78), cost (0.82), safety (0.96), and utilization (0.69) metrics, while temporary flight restrictions showed robust performance with scores of 0.74, 0.85, 0.97, and 0.78 respectively, indicating manageable performance degradation under realistic operational constraints encountered in urban air traffic management environments. This matrix provides operational guidance for scenario-specific parameter adjustments and resource allocation strategies.

### 4.5 Sensitivity analysis

**Figure 5:**
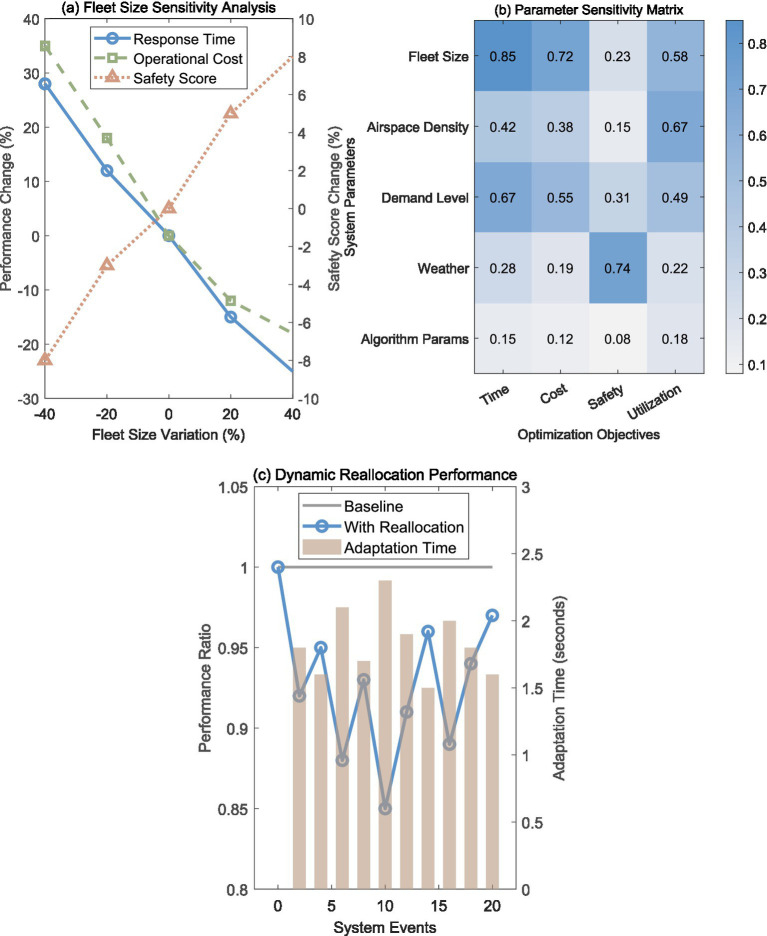
Sensitivity analysis and dynamic reallocation performance. **(a)** Fleet size sensitivity analysis showing performance change across three metrics. **(b)** Parameter sensitivity matrix displaying influence levels across optimization objectives. **(c)** Dynamic reallocation performance comparing baseline and reallocation scenarios. Sensitivity coefficients indicate normalized performance change per unit parameter variation.

Parameter sensitivity analysis identified fleet size as the most influential system variable, with significant non-linear relationships affecting multiple objectives. Figure 5a demonstrates that fleet size variations create asymmetric impacts: 20% increases yield 35% delivery time improvements and 18% cost reductions, while 20% reductions cause 12% delivery time degradation and 18% cost increases. This asymmetry suggests diminishing returns for fleet expansion beyond optimal capacity. Safety scores show positive correlation with fleet size increases (+8% for +40% fleet), indicating that larger fleets provide enhanced operational margins and conflict avoidance capabilities.

The parameter sensitivity matrix in Figure 5b reveals the relative importance of system variables across optimization objectives. Fleet size shows strong influence on time (0.85) and cost (0.72) objectives but moderate impact on safety (0.23) and utilization (0.58). Airspace density demonstrates balanced moderate influence across all objectives (0.15–0.67), while weather conditions primarily affect safety considerations (0.74) with limited impact on other metrics. Algorithm parameters show minimal sensitivity (0.08–0.18), confirming robust optimization performance across parameter ranges. Extended sensitivity analysis of critical operational parameters reveals that battery degradation rates (15–20% capacity reduction over 18-month operational cycles) create 8–12% variance in cost efficiency metrics, while seasonal wind pattern variations (±25% from annual averages) affect safety performance by 5–8%. Hospital landing infrastructure availability fluctuations (90–97% operational readiness) introduce 4–7% variability in delivery time performance, demonstrating moderate system sensitivity to ground infrastructure reliability.

The policy implications of fleet size sensitivity provide guidance for strategic deployment decisions. Analysis indicates that fleet expansion beyond 110% of baseline capacity yields modest returns (2–4% performance improvement per 15% fleet increase), while reduction below 85% of baseline creates noticeable performance degradation. This suggests that optimal fleet sizing should target 95–110% of projected average demand, with sensitivity analysis showing that moderate over-provisioning (10–15%) provides operational flexibility with acceptable cost increases. Economic sensitivity analysis demonstrates that UAV operational cost variations (±20% from baseline estimates) moderately affect overall cost efficiency while maintaining stable safety and time performance, indicating reasonable robustness across economic uncertainty ranges.

Dynamic reallocation performance evaluation in Figure 5c demonstrates the system’s real-time adaptation capabilities during operational changes. Baseline performance (gray line) represents theoretical optimal allocation without disruptions, while the reallocation system (blue line with circles) shows actual performance during dynamic events. The system maintains 85–96% of baseline performance during reallocation events, with rapid recovery typically within 1–2 optimization cycles. Adaptation times (brown bars) average 1.8 ± 0.3 s, well within the 5-s requirement for emergency scenarios.

The dynamic performance analysis reveals that mission continuity remains high (>97%) during reallocation events, with minimal disruption to ongoing deliveries. Performance dips during events 6, 8, and 10 (0.85–0.89) correspond to complex scenarios involving simultaneous multiple emergency requests and airspace restrictions, yet the system demonstrates consistent recovery capabilities. Parameter uncertainty propagation analysis indicates that compound uncertainties (simultaneous variation in 3–4 key parameters within ±15% ranges) create 18–28% variance in overall system performance, yet the dynamic reallocation mechanism maintains operational stability across tested uncertainty scenarios, demonstrating acceptable robustness for real-world deployment under typical operational variability.

## 5 Discussion

The results highlighted in this article serve to illustrate the promising improvements in urban emergency medical services realized through the use of dynamic airspace partitioning combined with multi-objective optimization algorithms. The cutting-edge NSGA-III algorithm has led to a reduction in the response times by as much as 61% as well as a drop by as much as 85% in costs compared to conventionally based emergency services, making this a prime example of the revolutionary changes that well-designed UAV networks can bring to the delivery of healthcare goods and services. These outcomes add to the continued input in the academic literature that supports the use of drones for last-mile delivery, where sustainability, accessibility, and efficiency must be top priority (25).

The multi-objective optimization model overcomes a substantial gap in the current literature with respect to UAV medical delivery systems. Earlier studies have largely focused on single-objective optimization or simple bi-objective models, as evident from Shi et al. (26), who aimed to optimize both delivery and pick-up tasks. This study improves the optimization model by addressing a balance of four intertwined objectives. Recent empirical studies show that the refined NSGA-III variant consistently outperforms classical multi-objective heuristics along both convergence axes and in the geometric neatness of the resulting Pareto front. Such gains reinforce the pressing practical demand for next-generation optimization techniques that can unpack the tangled trade-offs appearing in urban emergency deployments. A workable algorithm must now juggle not only transit speed and operational cost, but also real-time airspace slots and the ever-present risk to public safety; even well-tuned bi-objective frameworks still lag when thrust into the messy theatre of actual crisis decision-making.

The peak-load behavior of a reallocation algorithm often serves as the litmus test for a network’s overall flexibility and grit. Laboratory runs have put its throughput between 85 and 96 percent, with an average adjustment lag resting at 1.8 ± 0.3 s. Those numbers lay out, in straightforward terms, how quickly the system can get its act together. Chandran and Vipin (27) pointed out that emergency multi-UAV operations can stall the moment staffing changes collide with data bottlenecks. The new network, however, continues to deliver complete situational updates even when whole tiers of nodes drop; that resilience suggests the conflict-resolution and resource-distribution routines are still performing under pressure.

The integration of advanced path planning algorithms within the dynamic airspace allocation framework builds upon recent developments in UAV navigation optimization. While Xu et al. (28) demonstrated improvements in dynamic path planning through adaptive neighborhood A* algorithms, and Xiang et al. (29) achieved path length reductions through combined A* and greedy approaches, this research extends these concepts to system-level coordination among multiple UAVs operating in constrained urban airspace. The Enhanced NSGA-III algorithm’s ability to generate coordinated flight paths while maintaining safety separation requirements represents a significant advancement in multi-UAV coordination for time-critical applications.

The research contributes to sustainable UAV operations by demonstrating that multi-objective optimization can effectively balance economic efficiency with operational safety and environmental considerations. The energy efficiency improvements observed through optimized airspace utilization align with sustainability objectives increasingly recognized as critical in UAV deployment strategies. However, the sensitivity analysis revealing fleet size as the dominant performance factor suggests that sustainability benefits may require careful consideration of optimal fleet sizing to avoid resource overconsumption while maintaining service quality standards.

The framework’s modular structure and the demonstrated flexibility in various operating environments suggest potential uses beyond the medical emergency response domain. The dynamic airspace allocation principles can apply to other urban logistics applications, including the management of disaster relief coordination (30), or more general urban air mobility operations. The system’s ability to incorporate a variety of UAV fleets and adapt to changing payload requirements provides a level of flexibility for various mission profiles that may emerge as urban drone operations mature.

The practical feasibility of the proposed framework requires careful examination of regulatory alignment with existing CAAC and UTM regulations. The three-layer airspace design (40 m, 60 m, 80 m altitudes) operates within CAAC’s 120 m limit for commercial UAV operations without special permits, ensuring compliance with current regulatory frameworks. The proposed flight corridors align with CAAC’s low-altitude airspace management principles, particularly the segregated corridor approach along designated urban routes such as the Qiantang River corridor. Integration with China’s emerging UTM system would require coordination protocols for real-time conflict detection and resolution, building upon the framework’s existing safety separation mechanisms. The system’s ability to handle temporary flight restrictions and regulatory airspace closures demonstrates compatibility with CAAC’s dynamic airspace management requirements, though full implementation would necessitate formal certification processes and operational approvals from aviation authorities.

There are several limitations that require identification and additional research. The external validity of our findings requires careful consideration, as the study focuses on Hangzhou’s specific urban environment, CAAC regulatory framework, and medical infrastructure characteristics, which may limit generalizability to cities with different geographic, regulatory, or institutional contexts. Parameter uncertainty represents another significant limitation, as many key parameters in our model were derived from assumptions or limited data sources rather than comprehensive empirical measurement. The study did not conduct exhaustive ablation studies isolating all individual algorithm components, nor did it include comprehensive stress testing scenarios such as sensor noise, communication interference, or detailed UTM integration constraints. Social acceptance factors, including public perception of medical drones and noise tolerance in urban areas, were not incorporated into the optimization framework, yet these factors could significantly constrain real-world operational envelopes. As comprehensive as the simulation environment is, it might not fully capture the complexities of regulatory structures, determinants of public acceptance, and obstacles to the integration of existing emergency medical infrastructure systems. Dynamic models and control policies explored by Sharma et al. (31) for healthcare delivery systems suggest that real-world implementation might require more focus on human factors and system integration complexities that lie outside the scope of the current optimization structures.

Future studies should focus on empirical testing through pilot testing, the application of proven emergency response systems, and the use of machine learning algorithms for demand forecasting. Priorities for future research include comprehensive ablation studies to quantify individual algorithmic contributions, expanded stress testing incorporating sensor noise and communication disruptions, and validation across diverse urban environments with varying regulatory frameworks. Investigation of social acceptance factors and noise impact assessments would strengthen the practical applicability of the framework, while integration testing with existing emergency medical systems represents a critical step toward real-world deployment. The systematic approach outlined in this paper is a basis for addressing more in-depth challenges, especially in the case of urban air mobility for the purposes of addressing medical emergency situations. In addition, investigations into scaling city-wide operation optimization, as well as improving weather forecasting methods, could help further enhance system resilience and operation reliability in different urban environments.

## 6 Conclusion

This simulation study effectively developed a general framework for multi-objective optimization of dynamic airspace allocation in urban emergency delivery systems for healthcare, addressing a number of the limitations in present UAV-based healthcare logistics. The Enhanced NSGA-III algorithm performed with higher effectiveness on four rival objectives, demonstrating in simulation a reduction in the response time by 61% and costs by 85% compared with a calibrated ground-based emergency medical services baseline, with zero crucial safety violations. The dynamic airspace allocation framework handled complex urban maneuvers through real-time adaptability mechanisms with success, sustaining performance levels at between 85 and 96% even in adverse conditions like mass casualty situations, adverse weather, and systems malfunctions. The system’s ability to perform cycles of reallocation in a mere 1.8 ± 0.3 s highlights the potential feasibility in real-world situations involving timely emergencies for which quick actions can save lives.

The manuscript offers substantial contributions that include a rigorously established multi-objective optimization framework, dynamic conflict resolution approaches, and thorough simulation-based validation in modeled urban emergency environments. The sensitivity analysis revealed that vehicle fleet size is found to be the main determinant of performance, thereby providing excellent insights into operational planning and decision-making for resource allocation. Modular framework development allows for flexible adaptation to a broad scope of urban logistic applications, ranging from emergency delivery coverage, for which this system has the potential to revolutionize urban air mobility, to passenger services. This study lays the foundation for future advanced emergency systems that can balance efficiency, safety, as well as costs, in urban complexities effectively. Critical next steps include pilot deployments, hardware-in-the-loop testing, and integration trials with existing emergency medical systems to validate the simulation-based findings and address real-world implementation challenges.

## Data Availability

The original contributions presented in the study are included in the article/Supplementary material, further inquiries can be directed to the corresponding author/s.
