# Supplementary material for: Multi-objective optimization model for dynamic airspace allocation of urban drones in medical emergency delivery
Source: Front Public Health. 2026 Feb 12;13:1727647. doi: 10.3389/fpubh.2025.1727647 (PMC12935914; doi:10.3389/fpubh.2025.1727647)
Supplement: Supplementary file 1 [file Supplementary_file_1.docx]

# Appendix

## Appendix A: Mathematical Model Specification

### A.1 Sets and Symbol Definitions

**Table A1. Set Definitions**

| Set | Definition |
| --- | --- |
| $I$ | Emergency medical request set, indexed from 1 to \|I\| |
| $J$ | Airspace segment set, indexed from 1 to \|J\| |
| $K$ | UAV fleet set, indexed from 1 to \|K\| |
| $T$ | Discrete time period set, indexed from 1 to \|T\| |

**Table A2. Decision Variable Definitions**

| Variable | Definition | Domain | Unit |
| --- | --- | --- | --- |
| $x_{ijkt}$ | UAV $k$ serves request $i$ via segment $j$ at time $t$ | $0,1$ | - |
| $route_{kjj't}$ | UAV $k$ moves from segment $j$ to $j'$ at time $t$ | $0,1$ | - |
| $location_{kjt}$ | UAV $k$ is located at segment $j$ at time $t$ | $0,1$ | - |
| $y_{jt}$ | Airspace segment $j$ is activated at time $t$ | $0,1$ | - |
| $e_{kt}$ | Remaining battery energy of UAV $k$ at time $t$ | $[0,bat_{k}]$ | kWh |
| $z_{kt}$ | Instantaneous power consumption of UAV $k$ at time $t$ | $[0,power_{max,k}]$ | kW |
| $conflict_{kk't}$ | Conflict occurs between UAVs $k$ and $k'$ at time $t$ | $0,1$ | - |
| $s_{it}$ | Service for request $i$ starts at time $t$ | $0,1$ | - |
| $comp_{it}$ | Service for request $i$ completes at time $t$ | $0,1$ | - |

**Table A3. System Parameter Definitions**

| Parameter | Definition | Unit |
| --- | --- | --- |
| **Demand Characteristic Parameters** | | |
| $w_{i}$ | Priority weight of emergency request $i$ (1-5 scale) | - |
| $deadline_{i}$ | Latest completion time for request $i$ | minutes |
| $payload_{i}$ | Required payload weight for request $i$ | kg |
| $service_{t}ime_{ijk}$ | Service time for UAV $k$ to complete request $i$ at segment $j$ | minutes |
| **UAV Specification Parameters** | | |
| $bat_{k}$ | Maximum battery capacity of UAV $k$ | kWh |
|  | Operational cost per hour for UAV  | $/hour |
| $speed_{k}$ | Cruise speed of UAV $k$ | km/h |
| $cap_{p}ayload_{k}$ | Maximum payload capacity of UAV $k$ | kg |
| $power_{max,k}$ | Maximum power consumption of UAV $k$ | kW |
| $power_{base,k}$ | Base hovering power consumption of UAV $k$ | kW |
| $power_{flight,k}$ | Additional power consumption for movement of UAV $k$ | kW |
| $power_{payload,k}$ | Power consumption coefficient per unit payload for UAV $k$ | kW/kg |
| $power_{wind,k}$ | Wind resistance power consumption coefficient for UAV $k$ | kW/(m/s) |
| **Airspace Parameters** | | |
| $cap_{j}$ | Traffic capacity of airspace segment $j$ | UAVs |
|  | Opportunity cost of activating airspace segment  | $/hour |
| $adjacent_{jj'}$ | Binary indicator: segments $j$ and $j'$ are adjacent (1=adjacent, 0=not adjacent) | - |
| $distance_{jj'}$ | Distance between airspace segments $j$ and $j'$ | km |
| $weather_{r}isk_{jt}$ | Weather risk index for segment $j$ at time $t$ | - |
| $wind_{jt}$ | Wind speed at segment $j$ at time $t$ | m/s |
| $safe_{min}$ | Minimum safety separation distance | m |
| **System Parameters** | | |
| $\Delta t$ | Time discretization interval | seconds |
| **Objective Function Coefficients** | | |
|  | Late delivery penalty coefficient | $/minute |
|  | Energy cost coefficient | $/kWh |
| $\alpha_{1}$ | Airspace utilization reward coefficient | - |
| $\alpha_{2}$ | Activation waste penalty coefficient | - |
|  | Conflict penalty coefficient | $/conflict |
|  | Weather risk penalty coefficient | $/risk unit |
| $\beta_{4}$ | Operational complexity penalty coefficient | - |

### A.2 Objective Functions

**Delivery Time Minimization:**

**Operational Cost Minimization:** $\min f_{2}=\sum_{k\in K} \sum_{t\in T} c_{k}\cdot\max\left(1,\sum_{j\in J} location_{kjt}\right)+\alpha\sum_{k\in K} \sum_{t\in T} z_{kt}\cdot\Delta t+\sum_{j\in J} \sum_{t\in T} \gamma_{j}\cdot y_{jt}$

**Airspace Efficiency Maximization:** $\max f_{3}=\alpha_{1}\sum_{j\in J} \sum_{t\in T} \frac{\sum_{k\in K} \sum_{i\in I} x_{ijkt}\cdot service_{t}ime_{ijk}}{cap_{j}\cdot y_{jt}\cdot\Delta t}-\alpha_{2}\sum_{j\in J} \sum_{t\in T} y_{jt}\cdot\max\left(0,1-\frac{\sum_{k\in K} \sum_{i\in I} x_{ijkt}}{0.3\cdot cap_{j}}\right)$

**Safety Risk Minimization:** $\min f_{4}=\beta_{1}\sum_{k\in K} \sum_{k'\in K,k'\neq k} \sum_{t\in T} conflict_{kk't}+\beta_{3}\sum_{k\in K} \sum_{j\in J} \sum_{t\in T} location_{kjt}\cdot weather_{r}isk_{jt}+\beta_{4}\sum_{k\in K} \sum_{t\in T} \sum_{j\in J,j'\in J,j\neq j'} route_{kjj't}$

### A.3 Constraint System

**A.3.1 Path Continuity Constraints**

Location state transition constraint: $location_{kjt}-location_{kj,t-1}=\sum_{j'\in J} route_{kj'jt}-\sum_{j'\in J} route_{kjj't}, \forall k\in K,j\in J,t\in T\setminus1 (C1)$

Service-location consistency constraint:$\sum_{i\in I} x_{ijkt}\leq location_{kjt}, \forall k\in K,j\in J,t\in T (C2)$

Path adjacency feasibility constraint: $route_{kjj't}\leq adjacent_{jj'}, \forall k\in K,j\in J,j'\in J,t\in T (C3)$

Movement time constraint: $route_{kjj't}\cdot\frac{distance_{jj'}}{speed_{k}}\leq\Delta t, \forall k\in K,j\in J,j'\in J,t\in T (C4)$

**A.3.2 Assignment Constraints**

Unique request assignment constraint: $\sum_{j\in J} \sum_{k\in K} \sum_{t\in T} x_{ijkt}=1, \forall i\in I (C5)$

Service start uniqueness constraint: $\sum_{t\in T} s_{it}=1, \forall i\in I (C6)$

Service completion uniqueness constraint: $\sum_{t\in T} comp_{it}=1, \forall i\in I (C7)$

UAV location exclusivity constraint: $\sum_{j\in J} location_{kjt}\leq1, \forall k\in K,t\in T (C8)$

**A.3.3 Energy Constraints**

Battery state transition equation: $e_{k,t+1}=e_{kt}-z_{kt}\cdot\Delta t, \forall k\in K,t\in T\setminus|T| (C9)$

Instantaneous power consumption model: $z_{kt}=power_{base,k}\cdot\max\left(1,\sum_{j\in J} location_{kjt}\right)+power_{flight,k}\cdot\sum_{j\in J} \sum_{j'\in J} route_{kjj't}$ $+power_{payload,k}\cdot\sum_{i\in I} \sum_{j\in J} x_{ijkt}\cdot payload_{i}+power_{wind,k}\cdot\sum_{j\in J} location_{kjt}\cdot\max(0,wind_{jt}) (C10)$

Battery capacity constraint: $0.1\cdot bat_{k}\leq e_{kt}\leq bat_{k}, \forall k\in K,t\in T (C11)$

Initial battery state: $e_{k0}=bat_{k}, \forall k\in K (C12)$

**A.3.4 Capacity Constraints**

Airspace segment capacity constraint: $\sum_{i\in I} \sum_{k\in K} x_{ijkt}\leq cap_{j}\cdot y_{jt}, \forall j\in J,t\in T (C13)$

UAV payload capacity constraint: $\sum_{i\in I} x_{ijkt}\cdot payload_{i}\leq cap_{p}ayload_{k}, \forall k\in K,j\in J,t\in T (C14)$

**A.3.5 Safety Constraints**

Conflict detection constraint: $conflict_{kk't}\geq\sum_{j\in J} location_{kjt}\cdot location_{k'jt}-1, \forall k\in K,k'\in K,k\neq k',t\in T (C15)$

System conflict rate limitation: $\frac{\sum_{k\in K} \sum_{k'\in K,k'\neq k} \sum_{t\in T} conflict_{kk't}}{|K|\cdot(|K|-1)\cdot|T|}\leq0.001 (C16)$

**A.3.6 Temporal Logic Constraints**

Service start precedence constraint: $\sum_{t'\leq t} s_{it'}\geq\sum_{j\in J} \sum_{k\in K} \sum_{t'\leq t} x_{ijkt'}, \forall i\in I,t\in T (C17)$

Service completion postcondition constraint: $\sum_{t'=1}^{t} comp_{it'}\leq\sum_{t'=1}^{t-1} s_{it'}, \forall i\in I,t\in T\setminus1 (C18)$

### A.4 Model Characteristics and Complexity

**Model Scale:**

- Number of decision variables: $O(|I|\cdot|J|\cdot|K|\cdot|T|)$
- Number of constraints: $O(|K|\cdot|J|\cdot|T|+|I|\cdot|T|)$
- Objective functions: 4 competing objectives

**Time Discretization:**

- Time step size: $\Delta t=0.5$ seconds
- Planning horizon: 60-180 minutes depending on emergency scenario requirements

**Airspace Discretization:**

- Three-dimensional grid structure: $100 \times 100 \times 20$ meters
- Vertical stratification: Three altitude layers at 40m, 60m, and 80m

### A.5 Safety and Conflict Resolution Formal Specification

**Table A4. Safety Separation Requirements by Altitude Layer**

| Altitude Layer | Minimum Horizontal Separation | Minimum Vertical Separation | Detection Horizon |
| --- | --- | --- | --- |
| 40m layer | 50m | 15m | Immediate: 6 time steps, Short-term: 600 steps, Medium-term: 3600 steps |
| 60m layer | 75m | 15m | Immediate: 6 time steps, Short-term: 600 steps, Medium-term: 3600 steps |
| 80m layer | 100m | 20m | Immediate: 6 time steps, Short-term: 600 steps, Medium-term: 3600 steps |

**A.5.1 Conflict Detection Model**

Immediate conflict detection constraint (already defined in main model): $conflict_{kk't}\geq\sum_{j\in J} location_{kjt}\cdot location_{k'jt}-1, \forall k\neq k',t\in T (C19)$

Short-term conflict prediction for adjacent segments: $predict_{c}onflict_{kk',t+\Delta T}=\sum_{j,j'\in J} location_{kjt}\cdot route_{k'jj',t+\Delta T}\cdot\mathbf{1}distancejj'<safe_{min}, \forall k\neq k',t\in T (C20)$

Medium-term encounter probability based on planned routes: $encounter_{r}isk_{kk',t+T_{med}}=\sum_{j\in J} \sum_{t'=t}^{t+T_{med}} location_{kj,t'}\cdot location_{k'j,t'}\cdot weather_{r}isk_{jt'} (C21)$

**A.5.2 Priority-Based Resolution Framework**

UAV priority scoring for conflict resolution: $priority_{s}core_{kt}=\sum_{i\in I} \sum_{j\in J} w_{i}\cdot x_{ijkt}+\alpha_{battery}\cdot\frac{e_{kt}}{bat_{k}}+\alpha_{complexity}\cdot\sum_{j,j'\in J} route_{kjj't} (C22)$

Resolution precedence rule: $resolve_{o}rder_{kk't}=\mathbf{1}priority_{s}corekt>priority_{s}core_{k't} (C23)$

**A.5.3 Constraint Propagation to Optimization Algorithm**

Conflict-constrained offspring generation: $valid_{o}ffspring_{ijkt}=x_{ijkt}\cdot\left(1-\max_{k'\neq k}conflict_{kk't}\right) (C24)$

Safety-guided reference point adaptation: $safe_{r}eference_{w}eight_{r}=\frac{\sum_{solutions} safety_{c}ompliance_{r}}{\sum_{solutions} total_{s}olutions_{r}} (C25)$

where $safety_{c}ompliance_{r}$ counts solutions near reference point $r$ with zero conflicts.

**A.5.4 Temporal Resolution Strategies**

Speed adjustment constraint within UAV operational limits: $adjusted_{t}ravel_{kjj't}=\frac{distance_{jj'}}{speed_{k}\cdot speed_{f}actor_{kt}}, 0.7\leq speed_{f}actor_{kt}\leq1.3 (C26)$

Time slot shifting for conflict avoidance: $shift_{c}onstraint_{ijkt}=\sum_{t'=t-\Delta T_{shift}}^{t+\Delta T_{shift}} x_{ijkt'}\leq1, \forall i,j,k (C27)$

**A.5.5 Mission Delay Penalty Calculation**

Conflict-induced delay penalty:

Safety constraint violation penalty: $safety_{v}iolation_{kt}=\delta_{violation}\cdot\sum_{k'\neq k} conflict_{kk't}\cdot\left(1-\frac{distance_{current}}{safe_{min}}\right) (C29)$

**A.5.6 Performance Metrics for Safety Compliance**

System-wide conflict rate limitation:

$$\frac{\sum_{k\neq k'} \sum_{t\in T} conflict_{kk't}}{|K|\cdot(|K|-1)\cdot|T|}\leq\theta_{conflict}=0.001 (C30)$$

Separation loss rate per operational hour: $separation_{l}oss_{r}ate=\frac{\sum_{conflicts} duration_{conflict}}{\sum_{k,t} \max(1,\sum_{j} location_{kjt})\cdot\Delta t}\leq0.001\text{ per hour} (C31)$

Mission success rate under safety constraints: $mission_{s}uccess_{r}ate=\frac{\sum_{i} \mathbf{1}_{completed_{i}\wedge zero_{c}onflicts_{i}}}{|I|}\geq0.99 (C32)$

**A.5.7 Integration Parameters**

| Parameter | Definition | Value | Unit |
| --- | --- | --- | --- |
| $safe_{min}$ | Minimum safety separation distance | 50 | m |
| $T_{med}$ | Medium-term planning horizon | 3600 | time steps |
| $\Delta T_{shift}$ | Maximum time slot shifting range | 10 | time steps |
| $\alpha_{battery}$ | Battery priority weighting factor | 0.3 | - |
| $\alpha_{complexity}$ | Route complexity weighting factor | 0.2 | - |
|  | Safety violation penalty coefficient | 1000 | $/violation |
| $\theta_{conflict}$ | Maximum acceptable conflict rate | 0.001 | conflicts/operation |
